# Integrated single-nucleus sequencing and spatial architecture analysis identified distinct injured-proximal tubular types in calculi rats

**DOI:** 10.1186/s13578-023-01041-3

**Published:** 2023-05-19

**Authors:** Zhu Wang, Qiong Deng, Yanli Gu, Min Li, Yeda Chen, Jieyan Wang, Ying Zhang, Jianwen Zhang, Qiyi Hu, Shenping Zhang, Wei Chen, Zhenhua Chen, Jiaying Li, Xisheng Wang, Hui Liang

**Affiliations:** 1 Department of Urology, People’s Hospital of Longhua Shenzhen, Southern Medical University, Shenzhen, Guangdong 518109 P.R. China; 2 Central Laboratory, People’s Hospital of Longhua Shenzhen, Southern Medical University, Shenzhen, Guangdong 518109 P.R. China; 3 Department of Pathology, People’s Hospital of Longhua Shenzhen, Southern Medical University, Shenzhen, Guangdong 518109 P.R. China; 4 Department of Urology, The First Affiliated Hospital of Sun Yat-sen University, Guangzhou, Guangdong 518109 P.R. China; 5 Department of Urology, People’s Hospital of Longhua, Southern Medical University, 38 Jinglong Jianshe Road, Shenzhen, Guangdong 518109 P.R. China

**Keywords:** Urolithiasis, Single cell sequencing, Proximal tubular, Kidney stone, Spatial transcriptomics

## Abstract

**Background:**

Urolithiasis with high prevalence and recurrence rate, has impacts on kidney injury in patients, becomes a socioeconomic and healthcare problem in worldwide. However, the biology of kidney with crystal formation and proximal tubular injury remains essentially unclear. The present study aims to evaluate the cell biology and immune-communications in urolithiasis mediated kidney injury, to provide new insights in the kidney stone treatment and prevention.

**Results:**

We identified 3 distinct injured-proximal tubular cell types based on the differentially expression injury markers (Havcr1 and lcn2) and functional solute carriers (slc34a3, slc22a8, slc38a3 and slc7a13), and characterized 4 main immune cell types in kidney and one undefined cell population, where F13a1^+/high^/CD163^+/high^ monocyte & macrophage and Sirpa/Fcgr1a/Fcgr2a^+/high^ granulocyte were the most enriched. We performed intercellular crosstalk analysis based on the snRNA-seq data and explored the potential immunomodulation of calculi stone formation, and founded that the interaction between ligand Gas6 and its receptors (Gas6-Axl, Gas6-Mertk) was specifically observed in the injured-PT1 cells, but not injured-PT2 and -PT3 cells. The interaction of Ptn-Plxnb2 was only observed between the injured-PT3 cells and its receptor enriched cells.

**Conclusions:**

Present study comprehensively characterized the gene expression profile in the calculi rat kidney at single nucleus level, identified novel marker genes for all cell types of rat kidney, and determined 3 distinct sub-population of injured-PT clusters, as well as intercellular communication between injured-PTs and immune cells. Our collection of data provides a reliable resource and reference for studies on renal cell biology and kidney disease.

**Supplementary Information:**

The online version contains supplementary material available at 10.1186/s13578-023-01041-3.

## Background

Kidney stones or renal calculi, also known as nephrolithiasis or urolithiasis, are a major cause of morbidity and cause acute kidney injury (AKI) in patients with pain and urinary tract infection [1], leading to a high risk of end-stage renal disease [2]. The incidence of symptomatic kidney stones has increased in both the United States and China over the past decades [3, 4]. Kidney stones can recur at a rate of up to 52% within 10 years of the initial episode [5]. Considering the high prevalence and recurrence rate of kidney stones, they have become a socioeconomic and healthcare problem in many countries.

Kidney stones appear to be one of the oldest and most common diseases in medicine, and substantial advances have been made in recent years. However, the mechanism of kidney stone formation and development remains unclear. According to their mineralogical composition, kidney stones can be divided into five main types: calcium oxalate, carbonatite, urate, struvite, and brushite [6]. Approximately 80% of kidney stones are calcareous stones (calcium oxalate and/or calcium phosphate) [7, 8]. Particularly, calcium oxalate (CaOx) stone accounts for around 75% of the stone composition, and may contain different forms of crystals. Calcium oxalate monohydrate (COM) is the most common and most stable form of calculi [9].

The renal proximal tubule (PT) is the main workhorse for absorbing water, calcium, phosphates, and amino acids via a series of solute transporters and trans- or para-cellular pathways [10]. It can be divided into proximal convoluted tubule (PCT, S1–S2) and proximal straight tubule segments (PST, S3) with marked functional differences [11] and plays a critical role in the functional maintenance of kidney. It is also responsible for vitamin D metabolism [12, 13]. The PT injury is the most common type of renal involvement, which has attracted extensive attention for exploring a wide variety of diseases [14, 15]. Classically, the crystals were often formed in the distal tubule. However, PT epithelial cell injury, apoptosis, and inflammation could promote the adhesion and aggregation of crystals, with which related cellular processes and molecular functions remain unknown.

Recent technological advancements in RNA sequencing and spatial transcriptomics (ST) profiling have enabled the detailed characterization of the phenotypical and functional diversities of different cell populations [16–18], which is critical for understanding cellular communication. Previously, researchers conducted the scRNA-sequencing (scRNA-seq) of normal human kidneys and presented three subtypes of PT cells and two subtypes of collecting duct cells [19]. Ding et al. mapped and determined 13 cell types of the rat kidney from birth to maturity [20]. Karaiskos et al. characterized the gene expression in the glomerulus by scRNA-seq in health and disease, providing a comprehensive atlas of gene expression for the known glomerular cell types and potential subpopulations for endothelium and podocytes [21]. Lu et al. performed an scRNA-seq analysis of mouse mesangial cells and identified 173 genes specifically expressed in mesangial cells in glomeruli [22]. These studies indicated that scRNA-seq could be a powerful tool for molecular biology studies and provided reliable references for studies on renal cell biology and kidney disease. However, the mechanism of crystal formation and its mediated PT injury at the single-cell level is not completely explored. The kidney cell populations and gene expression profiles have never been characterized in a urolithiasis model at the single-cell level. In the present study, we performed integrated single nucleus RNA-sequencing (snRNA-seq) and spatial architecture analysis to identify and characterize distinct PT cell populations and cellular communications in the kidney of rats with calculi.

## Results

### Integrated single-nucleus RNA sequencing and spatial transcriptomics analysis on the kidneys of calculi rats

**Fig. 1:**
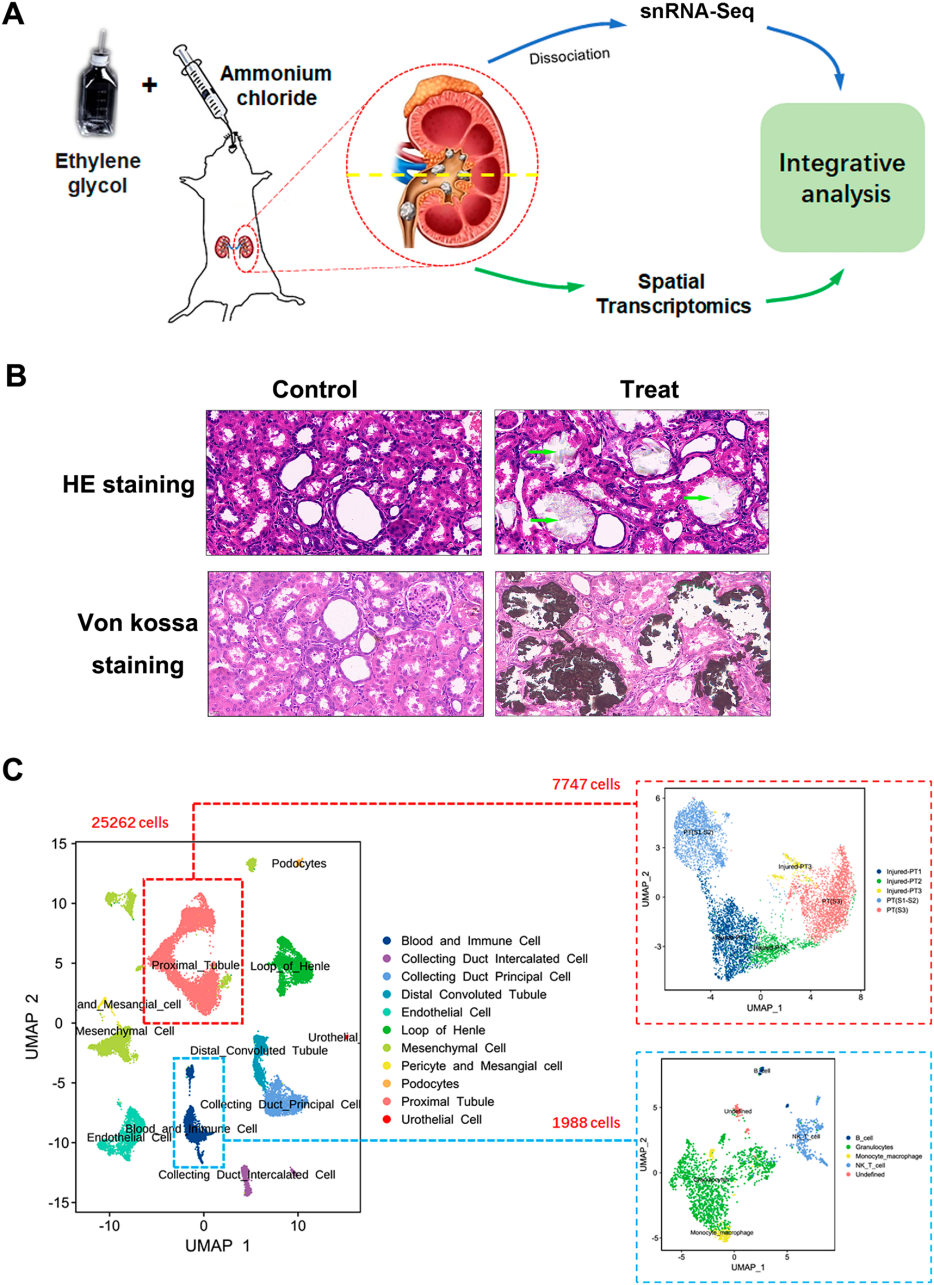
A single-cell transcriptomic analysis of rats with calculi. **A** Study design and workflow of rat kidney sample processing for snRNA-seq and spatial architecture analysis. **B** Histochemical validation and von Kossa staining of the calcium crystals in the urolithiasis model via HE staining; the green arrows indicate the crystals. Original magnification, 10 × 40. **C** Uniform manifold approximation and projection (UMAP) of snRNA-seq cells recovered from the kidneys of both normal rats and rats with calculi, as well as proximal tubular and BI subsets

A renal CaOx deposition rat model was successfully generated using ethylene glycol and ammonium chloride to mimic the kidney stone formation in humans. Renal site-matched samples of the rats were collected and prepared for integrated snRNA-seq and ST analysis (Fig. 1A). Prior to dissociation, the histopathologic examination, including HE and von Kossa staining was performed to detect CaOx deposits and tubulointerstitial damage of the rat kidney. Compared to the normal group, a large number of CaOx deposits were observed inside the proximal tubules, loops of Henle, distal tubules, and collecting ducts. Considerable tubulointerstitial damage, such as tubular atrophy, dilation, hyaline cast, tubular cell necrosis, and interstitial inflammation, was observed in the renal tissue of rats with calculi (Fig. 1B).

**Fig. 2:**
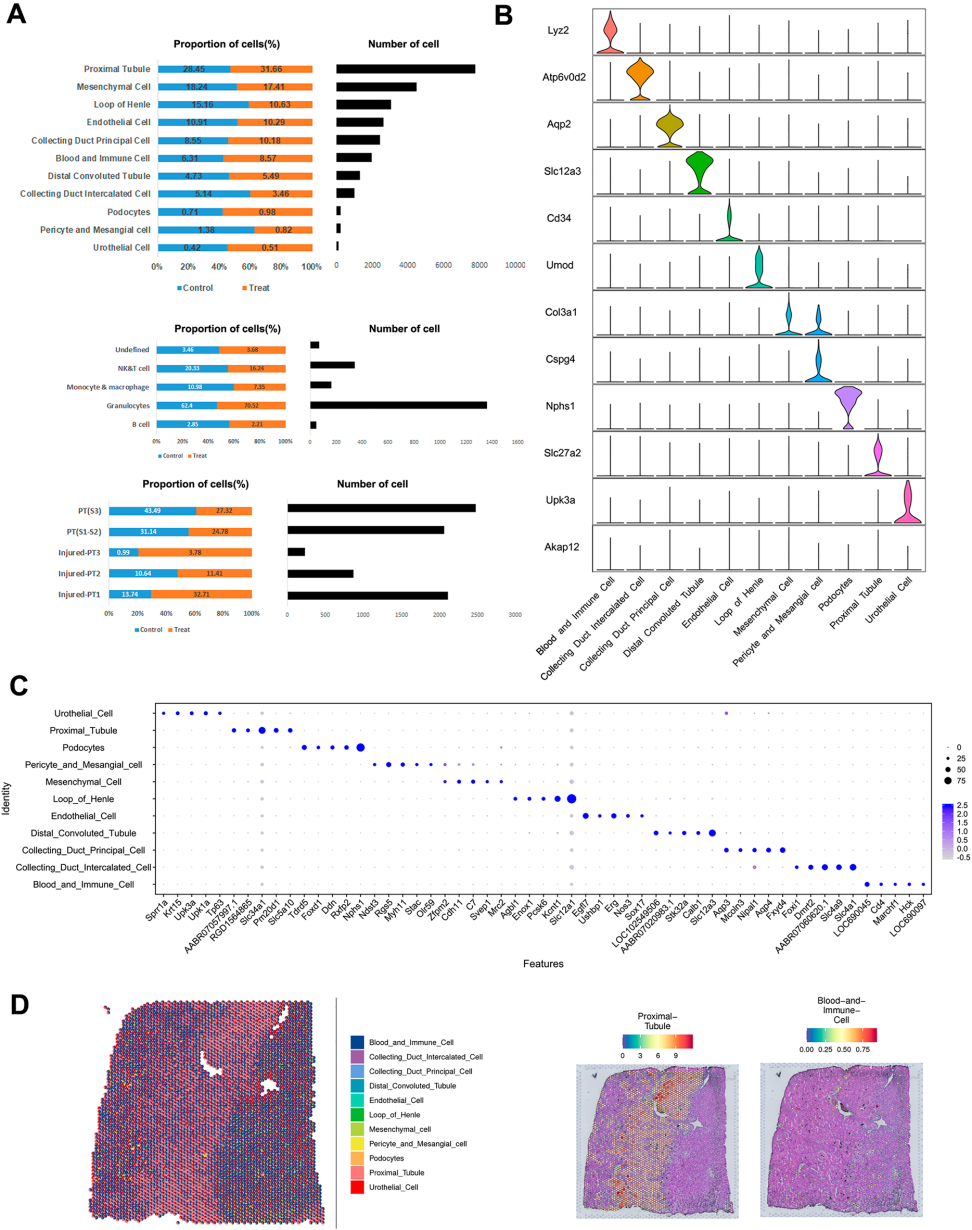
snRNA-seq revealed the distinct cell types in the kidneys of rats with calculi ** A** Bar plots of the proportion of cell types by origin and total cell number. **B** Distribution and relative expression of established marker genes (violin plots) for identifying the cell populations in rat kidneys. **C** Dot plot of top five cluster-specific genes of each cell type in the kidneys of rats. **D** Spatial feature plots of cell types identified in the kidney of calculi rats

We performed snRNA-seq analysis on the kidney of rats with calculi and normal controls using the 10× Genomics Chromium platform. A total of 15,262 qualified cells were assorted to 20 original clusters by t-distributed stochastic neighbor embedding (t-SNE) and uniform manifold approximation and projection (UMAP) analysis (Fig. 1C and Additional file 1: Fig. S1A). The cell-type-proportion analysis reflected consistent sample processing (Fig. 2A). The unsupervised clustering according to the curated marker genes from the literature identified 11 different cell types. The PT cluster included injured-PT1, injured-PT2, injured-PT3, PT (S1–S2), and PT (S3) subpopulations. The blood and immune (BI) cluster included B cells, granulocytes, monocytes and macrophages, natural killer (NK) and T cells, and undefined cell types. Besides the PT and BI, loop of Henle, pericytes, and mesangial cells, the distal convoluted tubule, collecting duct principal cells, collecting duct intercalated cells, podocytes, mesenchymal cells, endothelial cells, and urothelial cells were identified based on previously identified cell-type-specific markers and top differentially expressed genes (DEGs) (Fig. 2B, C). We next clustered the spatial transcriptomics data of the kidney of calculi rat. Unbiased clustering of each individual spatial detection spot resulted in 11 clusters. Cell-type score per spot showing the distribution of proximal tubular, blood and immune cells (Fig. 2D).

DEGs analysis showed that 2994 genes were altered in the bulk samples of kidney of rats with calculi, including 823 upregulated and 2171 downregulated genes. Notably, most DEGs were downregulated in all identified cell types in the kidneys of rats with calculi (Additional file 2: Fig. S2A). Kyoto Encyclopedia of Genes and Genomes (KEGG) pathway analysis showed that the DEGs were mainly enriched for metabolic pathways, followed by ribosome, endocytosis and oxidative phosphorylation (Additional file 2: Fig. S2B). Among of which, the 2-Oxocarboxylic acid metabolism has the highest rich factor (Additional file 2: Fig. S2C, D). We next annotated the DEGs in gene ontology (GO) including molecular function (MF), cellular component (CC), and biological process (BP). The DEGs in the treated group were significantly and abundantly enriched for cell, cell part and cellular process (Additional file 2: Fig. S2E). Gene set enrichment analysis (GESA) showed that ribosome, proteasome, systemic lupus erythematosus, cytokine-cytokine receptor interaction, IL-17 signaling pathway and staphylococcus aureus infection were the top 6 significant upregulated signaling pathways of the DEGs between calculi rat and control (Additional file 3: Fig. S3A). On the other hand, the glycerophospholipid metabolism, phosphatidylinositol signaling system, inositol phosphate metabolism, glyoxylate and dicarboxylate metabolism, ABC transporters and inflammatory mediator regulation of TRP channels were the top 6 significant downregulated signaling pathways (Additional file 3: Fig. S3B). Together, our integrative snRNA-seq analysis pointed to the cell types and DEGs in the kidney of calculi rats, defined a consistent and non-redundant catalog of cell types that comprise the distinct gene expression profiles and functions which provided a representative cell atlas on the kidney of nephrolithiasis.

### snRNA-seq revealed distinct injured-PT clusters in the kidney of calculi rats

**Fig. 3:**
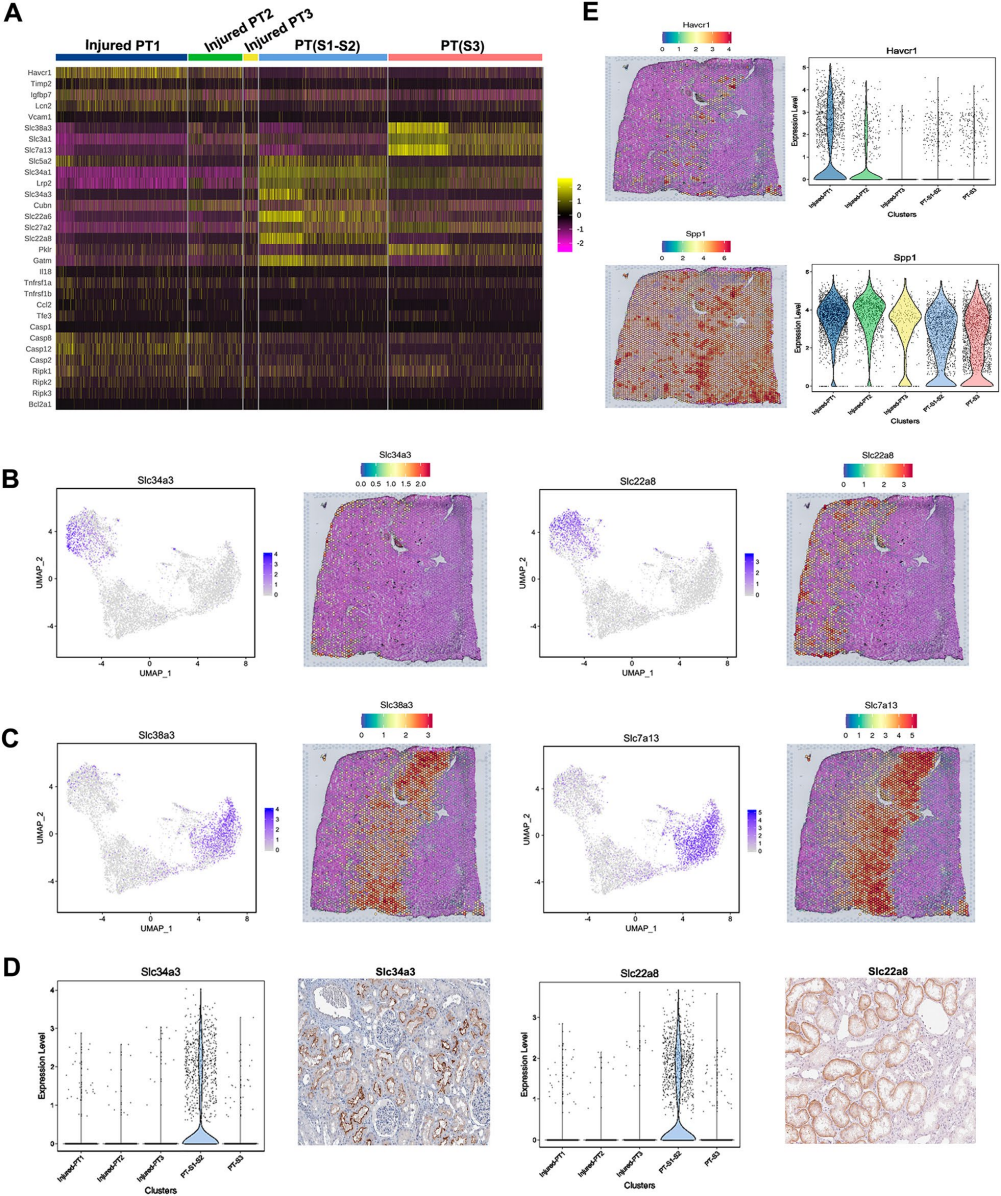
Single-cell RNA sequencing identified distinct proximal tubular populations in the kidneys of rats. **A** Heatmap of novel marker genes of subpopulations in proximal tubular. **B**, **C** UMAP snRNA-seq and spatial feature plots of marker genes expressed by proximal tubular subpopulations. **D** Violin plots of selected markers expression in distinct injured-proximal tubular cells. The representative immunohistochemistry images were adopted from the Human Protein Atlas (https://www.proteinatlas.org/). **E** Left: Spp1 and Havcr1 expression feature plots generated via ST platform; Right: Violin plots of Spp1 and Havcr1 in the kidney of calculi rats

PT cells play an important role in regulating systemic acid–base balance by controlling Na^+^-H^+^ and HCO^3−^ transport and have attracted extensive attention in the kidneys. After re-clustering of the PT cells by t-SNE and UMAP analysis (Fig. 1C, Additional file 4: Fig. S4A), we identified five major PT subpopulations, including renal proximal convoluted tubule (S1–S2) segment cells [PT(S1-S2)] based on the expression of markers slc34a3 and slc22a8 [23, 24], and proximal straight tubule (S3) segment cells [PT(S3)] based on the expression of known markers (slc38a3 and slc7a13) [25, 26] and DEGs (Fig. 3A-D, Additional file 4: Fig. S4B). The expression of these functional markers was consistent with previous scRNA-seq studies [25, 27].

Additionally, a distinct set of injured-PT cells were identified based on the partially or completely lost expression of solute carriers (slc22a12, slc38a3, slc7a12 and slc7a13) and increased expression of proximal tubular injury marker Spp1 (also known as Osteopontin, OPN), Lcn2 and Havcr1 (Fig. 3A, E).

**Fig. 4:**
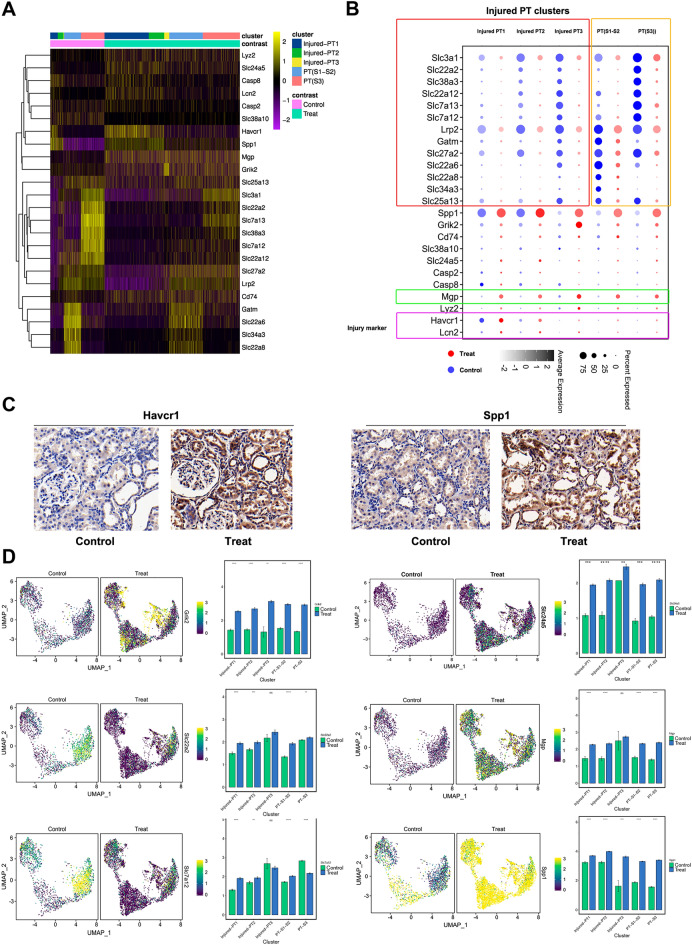
Profiling of distinct genes in the proximal tubular cells of rats with calculi. **A** Heatmap of functional genes and injury markers expressed in the proximal tubular cells. **B** Spot plot of functional genes and injury markers expressed in the proximal tubular cells. **C** Representative images of immunohistochemistry staining of injury markers (Havcr1 and Spp1) in the kidney of calculi rats. **D** Expression profiles of distinct markers in the proximal tubular cells of rats with calculi by UMAP and column plots. The gene expression level in different groups and sub-populations were showed in different plots with error bars. *, P < 0.05, **, P < 0.001; ***, P < 0.0001; ****, P < 0.00001; ns, non-significant

On comparing the DEGs of the proximal tubular cells between calculi rats and control, we found that 1394 genes were upregulated and 1979 genes were downregulated in the bulk proximal tubular cells (Additional file 5: Fig. S5A). Particularly, the transmembrane transporters Slc22a2 (OCT2), Slc38a3 (SNAT3), Slc7a13 (AGT-1), Slc7a12, Slc34a3, Slc22a8 (OAT3), and Slc22a6 (OAT1) were significantly decreased in the bulk proximal tubular cells of calculi rats, and the expression of Mgp, Spp1, Slc24a5, and Grik2 were increased as compared to the normal control (Fig. 4A, B). The kidney injury markers Havcr1 and Spp1 were significantly increased in the treated group. The expression at protein level was validated by immune-histochemistry staining (Fig. 4C). Interestingly, the increased expression of Grik2, Slc22a2, Mgp and Slc22a5 were observed in all the sub-clusters of proximal tubular cells. However, the Slc7a12 was only significantly decreased in the PT-S3 cluster (Fig. 4D).

**Fig. 5:**
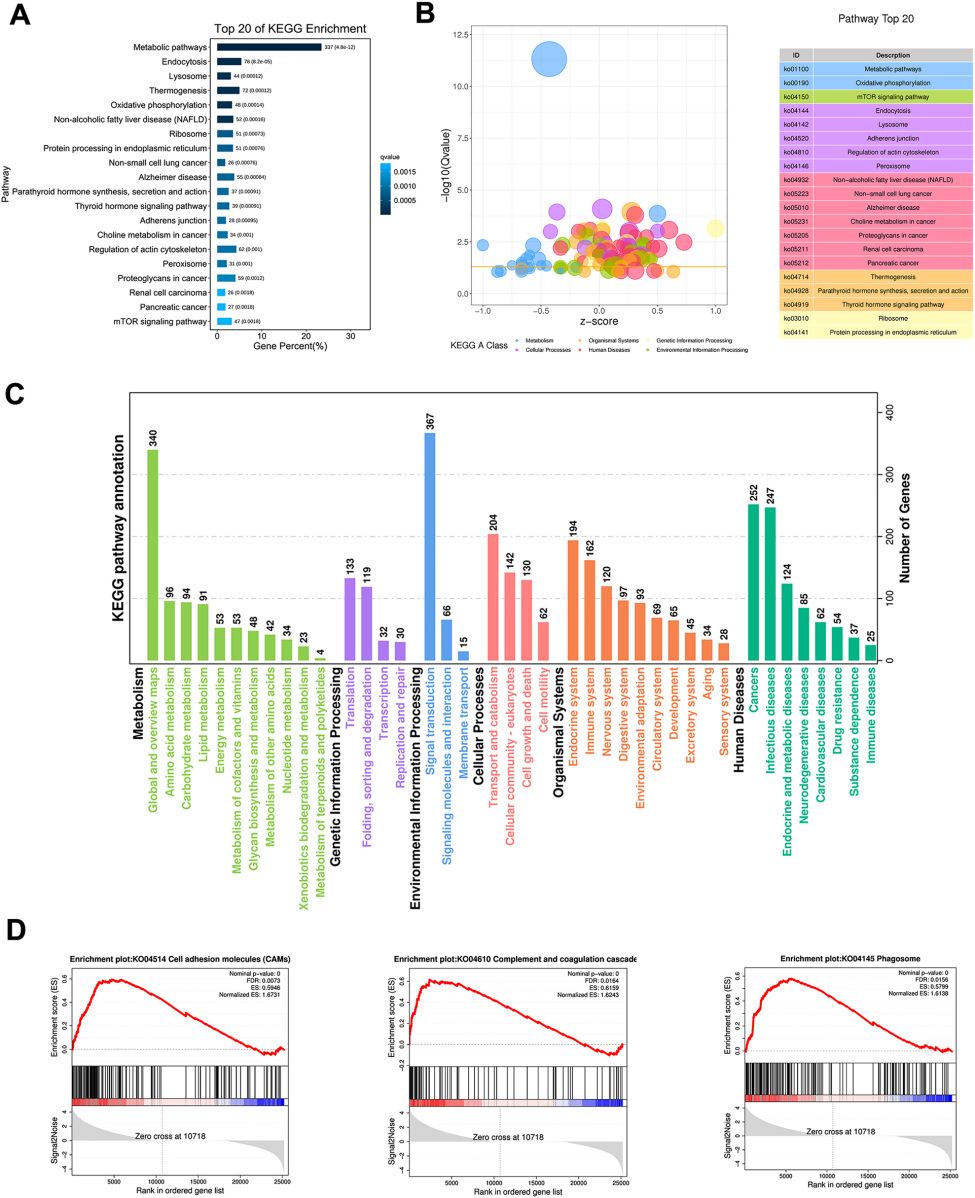
Biological significance of the DEGs in proximal tubular. **A** Bar plot of the top 20 KEEG signaling pathways enriched in the proximal tubular of rats with calculi. **B** Bubble plot of the top 20 KEEG signaling pathways enriched in the proximal tubular of rats with calculi. **C** KEGG pathway annotation in the proximal tubular of calculi rats. **D** The most significantly upregulated signaling pathways in the proximal tubular cells identified by GSEA.

The underlying biological functions and pathways of identified DEGs were predicted through KEGG enrichment analysis. A total of 337 DEGs facilitating the metabolic pathways were most enriched. The endocytosis and lysosomes were also highly enriched in the proximal tubular cells of rats with calculi (Fig. 5A, B and Additional file 5: Fig. S5B). KEGG pathway annotation indicated that (1) for metabolism, DEGs were particularly enriched in amino acid metabolism, carbohydrate metabolism, lipid metabolism and energy metabolism; (2) for environmental information processing, DEGs were enriched in signal transduction and signaling molecules and interaction; (3) for cellular process, DEGs were enriched in transport and catabolism, cellular community-eukaryotes, cell growth and death (Fig. 5C). KEGG enrichment analysis of sub-clusters of injured-proximal tubulars were also showed in Additional file 5: Fig. S5C-F, which indicated differences among different sub-clusters. Endocytosis, ribosome, lysosome and aminoacyl-tRNA biosynthesis were the most significantly enriched pathways in the cluster of injured-PT1 (Additional file 5: Fig. S5C). Metabolic pathways and endocytosis were the most enriched pathways in the cluster of injured-PT2 (Additional file 5: Fig. S5D). Most of the pathways were down-regulated in the cluster of injured-PT3 (Additional file 5: Fig. S5E, F).

GSEA analysis showed that the cell adhesion molecules, complement and coagulation cascades, and phagosomes were significantly enhanced in the proximal tubular cells of rats with calculi (Fig. 5D). In sum, these findings demonstrated three injured proximal tubular sub-populations with distinct gene expression profiles and signaling pathways in the kidney of calculi rats, which provided insights into the oxalate calculi stone related kidney injury.

### snRNA-seq revealed the immune landscape of kidneys in rats with calculi

**Fig. 6:**
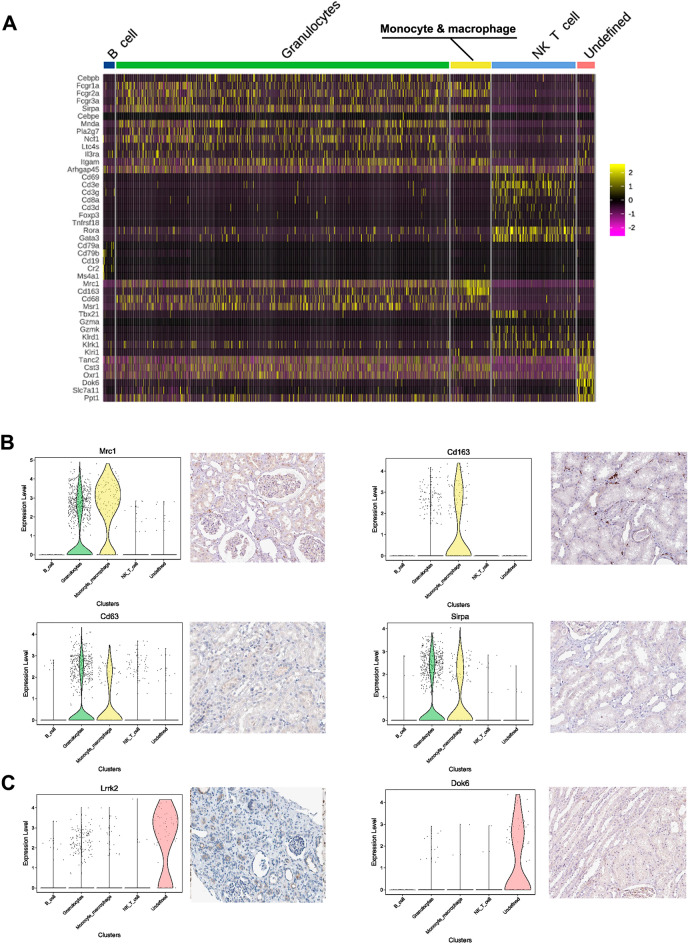
snRNA-seq revealed the immune landscape of kidneys of rats with calculi. **A** Heatmap of novel markers expressed in the BI cell sub-clusters. **B** Monocyte & Macrophage and Granulocyte populations were identified through the expression of Mrc1, CD163, CD63 and Sirpa. **C** An undefined cell population in the BI cluster was identified with high expression of Lrrk2 and Dok2. The representative immunohistochemistry images were adopted from the Human Protein Atlas (https://www.proteinatlas.org/)

We applied unsupervised re-clustering on the pool of experimental and control rat kidney samples, and identified eight subpopulations of the BI cells and re-clustered them by UMAP and t-SNE analysis (Fig. 1C, Additional file 6: Fig. S6A). The most abundant cell population comprised granulocytes and NK and T cells, followed by monocytes and macrophages (Fig. 6A and Additional file 6: Fig. S6B). The monocyte & macrophage cluster was identified by the expression of hemoglobin scavenger receptor CD163 and the mannose receptor CD206 (also known as Mrc1), which are both expressed on the surface of macrophages and correlated with antigen processing and presentation [28–30] (Fig. 6B, Additional file 6: Fig. S6C). Granulocytes (includes eosinophils, basophils, and neutrophils) is the major type of white blood cells and part of the innate immune system against bacterial infection. Fcgr1a (CD64), Fcgr2a (CD32a), and Fcgr3a (CD16a) are neutrophil markers highly expressed in the granulocyte cluster [31]. CD63, as a marker of basophil activation, is widely used in the basophil activation test in the diagnosis and monitoring of allergic diseases [32]. Which expression is enhanced in the granulocyte, monocyte, and macrophage clusters (Fig. 6B). Sirpa, a cellular ligand for CD47, is also selectively expressed in certain cell types [33], including macrophages and granulocytes (Fig. 6B). In addition, we also identified an undefined cluster of BI with high expression of Lrrk2 and Dok6 (Fig. 6C). Lrrk2 gene is a member of the leucine-rich repeat kinase family, presented largely in the cytoplasm but also associates with the mitochondrial outer membrane, recognized as a target for modulating immune system responses [34]. Downstream of tyrosine kinase 6 (DOK6) is a member of the DOK family of intracellular adaptors that play a role in the RET signaling cascade [35]. However, the role of these marker genes and such populations of BI clusters in the kidney of calculi rats is still not quite clear.

**Fig. 7:**
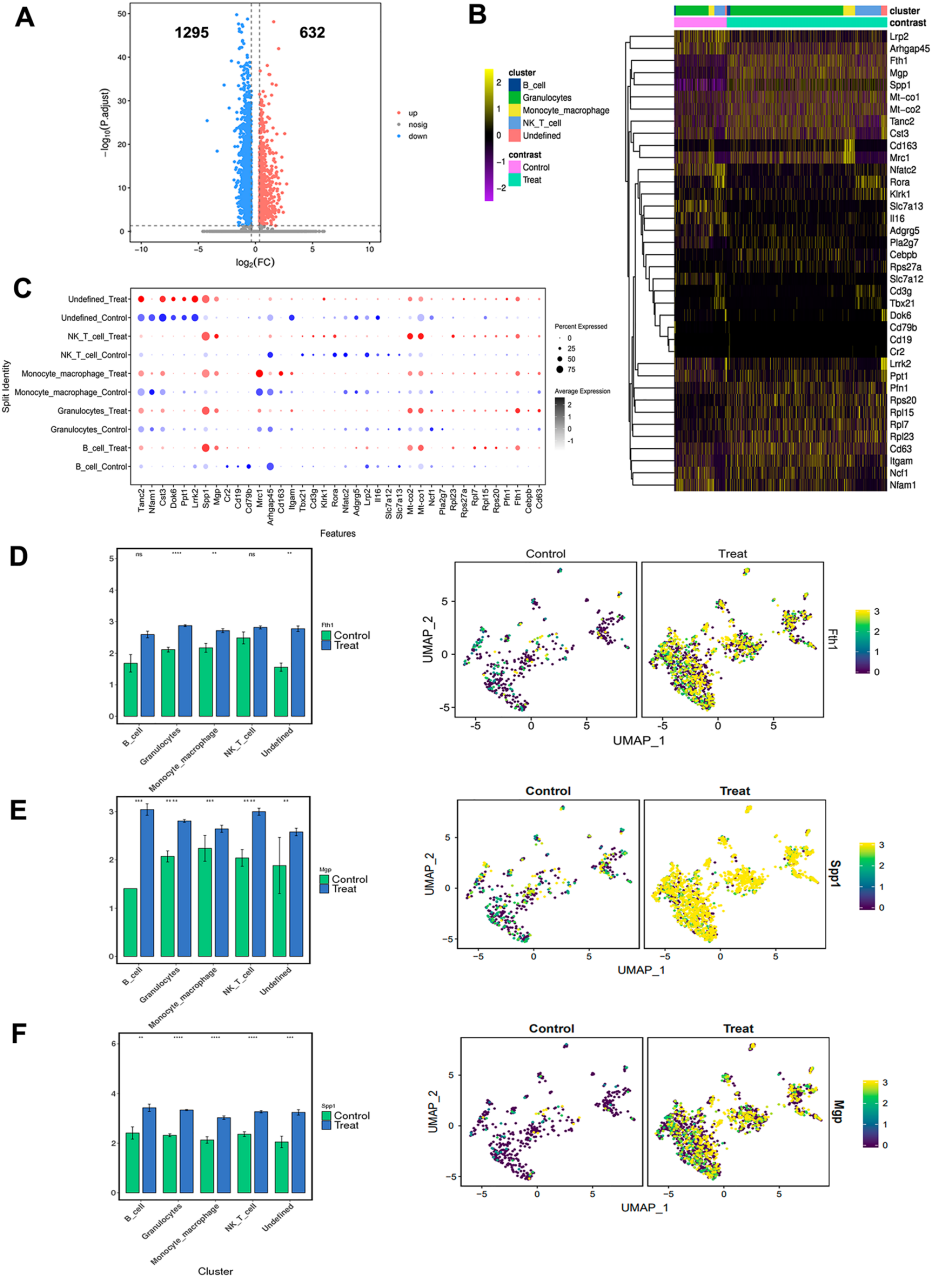
Profiles of gene expression in the BI cluster in the kidneys of rats with calculi. **A** Scatter plot of DEGs of the BI cluster in the kidneys of rats with calculi versus normal control. **B** Heatmap of distinct markers expression in the kidneys of rats with calculi versus normal control. **C** Dot plot of distinct novel marker expression in the BI cluster in the kidneys of rats with calculi versus normal control. **D** Column plot and UMAP analysis of Fth1. **E** Column plot and UMAP analysis of Spp1. **F** Column plot and UMAP analysis of Mgp. *, P < 0.05, **, P < 0.001; ***, P < 0.0001; ****, P < 0.00001; ns, non-significant

On comparing the DEGs of BI population in the kidney of control and calculi rats. We found that 1295 genes were downregulated and 632 genes were upregulated in the BI cluster (Fig. 7A). Particularly, the Fth1 (ferritin heavy chain 1, a marker of ferroptosis), matrix Gla protein (MGP), and Spp1 was significantly enhanced expression in the BI cluster in kidney of calculi rats (Fig. 7B–E).

KEGG enrichment analysis showed that (1) for cellular processes, DEGs were most enriched in endocytosis, lysosome, regulation of actin cytoskeleton and cellular senescence; (2) for DEGs were most enriched in Fc gamma R − mediated phagocytosis, osteoclast differentiation, natural killer cell mediated cytotoxicity, Th17 cell differentiation, B cell receptor signaling pathway, neurotrophin signaling pathway, T cell receptor signaling pathway, Th1 and Th2 cell differentiation (Additional file 7: Fig. S7A, B). Particularly, GSEA analysis showed that the cell cycle, proteasomes, and ribosomes were significantly upregulated in the BI population in kidney of calculi rats (Additional file 7: Fig. S7C). These findings demonstrated the immune landscape of the kidney of calculi rats, provide an insight for immune-regulatory investigation in kidney stone formation and related kidney injury.

### snRNA-seq identified novel interactions between proximal tubular and immune cells

**Fig. 8:**
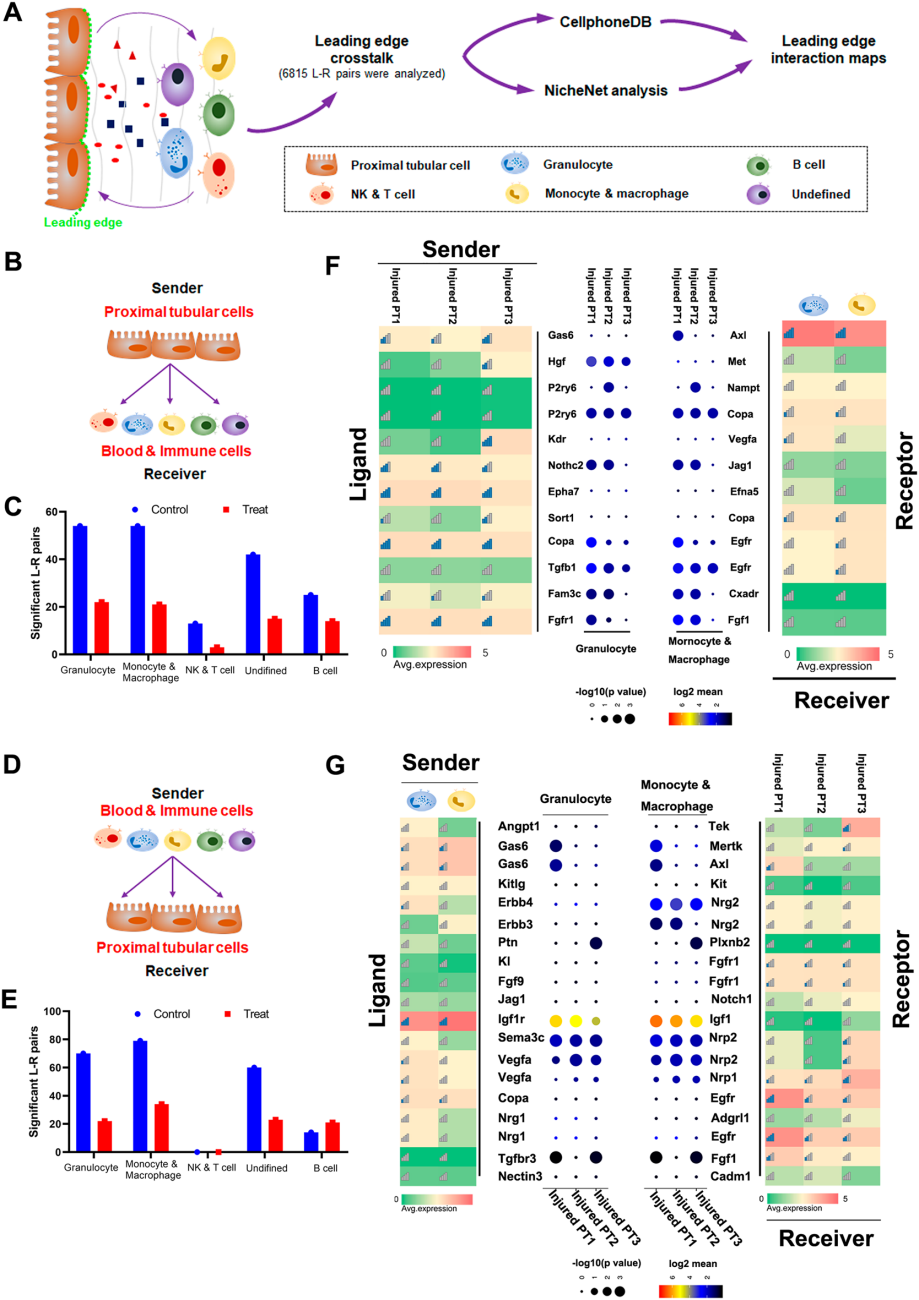
Novel interactions between injured-proximal tubular and immune cells. **A** Schematic of ligand–receptor interaction in injured-PT subpopulations at the leading edge and in immune cells. **B** and **C** Bar plots of significant ligand–receptor (L-R) pairs (*P* < 0.05) when PTs expressed ligands and immune cells expressed receptors matched to the cell types in snRNA-seq data. **D** Left: heatmap of snRNA-seq average log fold change (logFC) of NicheNet top predicted ligands expressed by injured-PT cells that modulated immune cell types. Middle: heatmap of significant L-R pairs between injured-PT subpopulations and immune cell types in snRNA-seq. Bottom: heatmap of snRNA-seq average logFC of ligand-matched receptors expressed by immune cell types. **E** and **F** Bar plots of significant L-R pairs (*P* < 0.05) when monocytes and macrophages and granulocytes expressed ligands and injured-PTs expressed receptors matched to cell types in snRNA-seq data. **G** Left: heatmap of snRNA-seq average log fold change (logFC) of NicheNet top predicted ligands expressed by monocyte and macrophages and granulocytes that modulated injured-PT cells. Middle: heatmap of significant ligand–receptor pairs between immune cell types and injured-PT subpopulations pair in snRNA-seq. Bottom: heatmap of snRNA-seq average logFC of ligand-matched receptors expressed by injured-PT subpopulations

Next, we applied Cellphone DB and NicheNet analyses to prioritize injured-PT cell ligands predicted to interact with immune cell-type signatures based on ligand–receptor (L-R) pairs (6815 L-R pairs) at the leading edge (Fig. 8A). Rats with calculi expressed much fewer ligands or receptors compared with the control rats (Fig. 8B-E). We paired the injured-PT cells enriched with ligands to the granulocytes and monocytes and macrophages enriched for the corresponding receptors (Fig. 8B). We found that the injured-PT cells and the granulocytes interacted via growth factors (Hgf, Tgfb1, and Fgr1), G-protein-coupled receptor (P2ry6), and FAM3 metabolism regulating signaling molecule C (Fam3c) (Fig. 8F). These interactions were also observed between the injured-PT cells and monocytes and macrophages. Hgf–Met interaction was identified only between injured-PT cells and granulocytes. We found that the injured-PT1 cells specifically interacted with monocytes and macrophages via GAS6–Axl signaling, as the injured-PT1 cells highly expressed Gas6, while the monocytes and macrophages expressed its receptor Axl.

Conversely, we paired the granulocytes and monocytes and macrophages enriched with ligands to injured-PT cells enriched for the corresponding receptors (Fig. 8D). We found that both granulocytes and monocytes and macrophages interacted with injured-PT cells via growth factors (Igf1r, Vegfa, Tgfbr3, and Ptn) and GAS6–Axl signaling. The interaction between ligand Gas6 and its receptors (Gas6–Axl and Gas6– MerTK) was specifically observed in the injured-PT1 cells, but not in injured-PT2 and injured-PT3 cells. The Ptn–Plxnb2 interaction was observed only between the injured-PT3 cells and its receptor-enriched cells (Fig. 8G). However, further investigation is required to understand whether this crosstalk reduction and differential expression profile are due to the intrinsic response of calculus crystal formation or immune-modulation activity.

## Discussion

Significant progress has been achieved in recent years in characterizing the kidney in development, homeostasis, fibrosis, acute kidney injury (AKI) and cancer at single cell level by using single-cell RNA sequencing (scRNA-seq) [20, 27, 36]. It is also reported that snRNA-seq provides comparable gene detection with reduced dissociation bias compare to scRNA-seq in adult kidney [18]. In present study, we conducted snRNA-seq and spatial architecture analysis to comprehensively characterize the gene expression patterns of all renal cell types in rats with ethylene glycol– and ammonium chloride–induced calculi, thereby providing a rich resource for further studies.

First, we identified three distinct injured-PT cell populations with the typical gene expression pattern of functional solute transporters and kidney injury markers. The well-known biomarker of kidney damage Havcr1, also known as kidney injury molecule-1 (KIM-1), is commonly elevated expression in AKI, renal trauma and kidney stone disease [27]. The Spp1 (also known as Osteopontin, OPN), which extensively upregulated in tubular, stromal, and immune cell in the murine model of AKI [27], was significantly increased in the proximal tubular and immune cells in the kidney of calculi rats as compare to the control group. Recent studies emphasized that renal tubular injury is a key mediator of the renal diseases and the final common pathway leading to the end-stage renal failure (ESRD) [37, 38], therefore, biomarkers reflecting renal tubular injury have been extensively explored [38, 39]. Interestingly, we found that the glutamate receptor 6 gene (Grik2), also known as GluR6, had significantly increased expression in the injured-PT3 cluster compared with the other two clusters of injured-PT cells, which might serve as a specific marker of this distinct injured-PT3 cell population. Previous studies indicated that Grik2 played a role in the maintenance of cancer stem cells by promoting sphere-forming ability, invasion ability, and tumorigenicity [40], and governed the cell proliferation of normal human fibroblasts [41]. However, the functional role of Grik2 in the proximal tubular cells and kidney stone related PTs injury remains unclear.

Previous studies of kidney stone models have mainly focused on the DEGs and related signaling pathways at cell-lines based level or in whole-kidney samples [42–45]. The present study firstly examined the effects of kidney stone on different cell types. Compared to the injured-PT1 and injured PT-2 clusters, the injured-PT3 population had relatively higher levels of solute transporters (Slc22a6, Slc7a12, and Slc22a12) and lower levels of injury markers, which might reserve the partial function of solute transporters and represent the primary stage of COM crystal–meditated proximal tubular injury. The three distinct injured-PT cell populations seem to represent the chronological order of appearance of the injured clusters in the rat model, which highlight different molecular processes in kidney stone formation due to a setting of constant ongoing injury in kidney of calculi rats.

Second, a comparison of our snRNA-seq data with bulk transcriptomes between treated and control rats revealed an apparent downregulation of metabolic pathways, including amino acid metabolism, carbohydrate metabolism, lipid metabolism and energy metabolism, which may responsible for the proximal tubular injury and tubular transcriptome alterations due to the high metabolic demands of proximal tubular cells becoming overwhelmed in the setting of ischemia or hypoxia [27, 36, 46, 47], similar to what were demonstrated in the mouse AKI models. Additionally, besides metabolic pathways, we also sorted out a series of DEGs related to ribosome, endocytosis and oxidative phosphorylation in bulk samples between treated and control rats. The processes of endocytosis of crystals, is a process that related to changes in the special components of the cytoskeleton, promoting the detention of crystals in the nephron, is an important factor of deposition of calcium in the kidney [48]. The oxidative phosphorylation is the main reactions to generate cellular adenosine triphosphate (ATP) in mitochondria [49]. A consequence of mitochondrial oxidative phosphorylation is the generation of reactive oxygen species (ROS) as a byproduct of mitochondrial respiration, which contributes to both homeostatic signaling as well as oxidative stress during pathology [49, 50]. The ROS related oxidative injury of renal tubular epithelial cell is a casual and essential factor in kidney oxalate calcium stone formation [51, 52].

In addition to the bulk transcriptome analysis of kidney in calculi rat, we also performed DEGs and signaling pathways enrichment analysis in proximal tubular cells between the control and treated group. Cell adhesion molecules (CAMs), complement and coagulation cascades, and phagosome were the most significant up-regulated pathways identified in the proximal tubular population via GSEA. Previous studies reported that complement and coagulation cascades activation leads to chemotaxis and immune-complex clearance [53], is one of the most significantly enriched signaling pathways in the calcium oxalate crystal-induced ROS in kidney [54]. The deposition of locally produced and activated complement fragments can also drive severe inflammatory response in the kidney and result in complement-mediated inflammatory injury [53]. Thus, these enhanced pathways could synergically promote the formation of kidney stone formation and its related proximal tubular injury.

Finally, we analyzed receptor-ligand pairs in a cell-specific manner, and identified distinct intercellular communications between injured-PT cells and immune cells in the kidneys of rats with calculi. In the present study, we identified four main immune cell types and one undefined cell population in the kidney of calculi rat. F13a1^+/high^/CD163^+/high^ monocytes & macrophages and Sirpa/Fcgr1a/Fcgr2a^+/high^ granulocytes were the most enriched immune sub-populations. Although the functional role of monocytes & macrophages in renal crystal initiation and development has been well characterized in previous studies [55–57], the crosstalk between the monocytes & macrophages and injured-PTs has never been investigated. In present study, we performed crosstalk analysis based on the snRNA-seq data and explored the potential immunomodulation of stone formation, and found that the interaction between ligand Gas6 and its receptors (Gas6–Axl and Gas6–MerTK) was specifically observed in the injured-PT1 cells, but not in injured-PT2 and -PT3 cells. Axl and MerTK are members of the TAM family of receptor tyrosine kinases, which are immune factors shared a common ligand growth arrest–specific protein 6 (Gas6) [58]. The interaction between MerTK and its ligand Gas6 involved in anti-inflammatory responses and designated to the clearance of apoptotic cells (ACs), was reported to play critical role in macrophage polarization [59, 60]. Axl is considered as a marker of M1 macrophage activation [61], and the Gas6–Axl signaling pathway is responsible for the activation of M1 macrophages and the polarization of M2 macrophages [62, 63]. Therefore, our data provided indications that the Gas6–Axl and Gas6–MerTK signaling might also play critical roles in the communication between injured-PT cells and monocytes & macrophages, contribute to the oxalate calculi stone initiation and development.

However, despite the comprehensive description of gene expression profiles, distinct sub-populations and intercellular communications in the kidney of calculi rats, we acknowledge several limitations of this study. First, due to the boundedness of high-throughput snRNA-seq and bioinformatic analysis, potential bias or imprecision may limit the confidence and generalizability of the findings. Further study and investment are warranted for experimental validation and underlying mechanism analysis to understand whether the differential expression profiles and distinct intercellular communications are due to the intrinsic response of calculus crystal formation or immune-modulation activity. Secondly, due to animal-based modeling and a limited observation timeframe, which could not perfectly mimic the status of crystals in the kidney of patients. Kidney organoids derived from human pluripotent stem cells might have potential utility for kidney stone modeling and further investigation.

## Conclusions

The present study comprehensively characterized the oxalate calculi stone related gene expression profiles and kidney injury landscape at the single-cell level. We identified novel marker genes and determined three distinct subpopulations of proximal tubular cells. We found that the distinct cell crosstalk between monocytes & macrophages and injured-PTs, particularly those interactions related to macrophage polarization and immune responses. These data collectively provided new insights into the pathophysiology of kidney stone formation and indicates injury-associated responses in different types of proximal tubular epithelial cells.

## Methods

### Development of the CaOx crystal rat model

All rat experiments were conducted according to the Guide for the Care and Use of Laboratory Animals prepared by the Institute of Laboratory Animal Resources for the National Research Council. The study was approved by the ethics committee of the People’s Hospital of Longhua, Shenzhen. Male Sprague–Dawley rats, weighing 225–250 g and purchased from Guangdong Medical Laboratory Animal Center, were maintained for at least 5 days under standard conditions with water and rat chow *ad libitum* and a 16-h light, 8-h dark cycle. We established the CaOx crystal rat model following the reported protocol [64]. The bilateral kidneys were removed and sent for further analysis. The cDNA libraries were sequenced on the Illumina sequencing platform by Genedenovo Biotechnology Co., Ltd. (Guangzhou, China).

### Sample processing and sequencing

The nuclei of above samples were isolated using iodoxanol density gradient centrifugation, resuspended in nucleus wash buffer, filtered through a 40-µm cell strainer, and measured. The gel beads-in-emulsion (GEM) generation and barcoding was performed as described previously [65]. About 10,000 nuclei per sample were loaded into a chromium single-cell 3′ chip (V3, 10x Genomics) following the manufacturer’s instructions. Silane magnetic beads (10× Genomics, PN-2,000,048) were used to remove the leftover biochemical reagents and primers from the post-GEM reaction mixture. After polymerase chain reaction (PCR) amplification, cDNA was cleaned up using an SPRIselect Reagent Kit (Beckman Coulter B23318). cDNA quality control and quantification were performed on an Agilent Bioanalyzer high-sensitivity chip. An ABI StepOnePlus Real-Time PCR System (Life Technologies) was used for quantitative analysis and pooling. The sequencing was performed according to the PE150 mode of Novaseq 6000. The raw sequencing data were processed using a cell ranger (10× Genomics, version 3.0.2) and aligned to the rat reference genome (Ensembl_release100.Rnor_6.0).

### snRNA sequencing data processing and analysis

The clusters were visualized using the uniform manifold approximation and projection (UMAP). The cell types were characterized based on the expression of known markers in the Cell Marker database and reported studies. The gene set enrichment analysis was performed with the R package fgsea with default parameters. The genes were ranked within clusters by multiplying avg_logFC by –log_10_(p_val) obtained from comparing stone and control samples with the FindMarkers function in Seurat. We used a draft network introduced by Ramilowski et al. to study ligand–receptor interactions [66] between the injured proximal tubular and blood and immune cell types. The detailed parameters of bioinformatic analysis were elaborated in the Additional file 8.

### Sample preparation and spatial transcriptomics

The embedded tissue blocks were cryosectioned in a cryostat to generate 10-µm sections for Visium Spatial slides while keeping the samples frozen. A Visium Spatial Tissue Optimization Slide Kit (10× Genomics, PN-1,000,191) was used to fit the time for permeabilization by generating fluorescently labeled cDNA tissue prints according to the manufacturer’s instructions. The spatially barcoded, full-length cDNA was amplified via PCR to generate sufficient mass for library construction. An ABI StepOnePlus Real-Time PCR System (Life Technologies) was used for quantitative analysis and pooling. The sequencing was performed according to the PE150 mode of Novaseq 6000. Details of sample preparation and spatial transcriptomics were described in the Additional file 8.

### Spatial transcriptomics data processing and integrative analysis

Raw sequencing data were processed using Space Ranger (10× Genomics) and aligned to the rat reference genome (Ensembl_release100.Rnor_6.0). Raw data, tissue staining, and chip serial numbers were input into Space Ranger for data quality control and sequence alignment to obtain high-quality sequencing data, the spatial distribution of spots in the tissue, and the expression of genes in each spot. Data were subjected to log homogenization, and PCA was used to reduce the variables. The spots were clustered and classified by the graph-based clustering algorithm. They were visualized using t-SNE and mapped in the tissue according to the staining result to view the distribution of each subgroup. Based on the cell types identified in single-nuclei RNA sequencing, the cells in each spot were classified using the R package MuSiC [67]. The cell composition proportion and tissue regions were visualized using Circos. The spatial distribution characteristics of the key genes in the tissues were intuitively shown by tissue mapping. The detailed methods of sample processing, sequencing and bioinformatic analysis were elaborated in the Additional files.

### Hematoxylin-eosin, von Kossa and immune-histochemistry staining

The kidneys were fixed with 4% paraformaldehyde in PBS, embedded in paraffin, and sectioned at 5-µm intervals, then dewaxed with acetate and dehydrated in ethanol and stained with hematoxylin-eosin (HE) or Von Kossa regents as described previously [68, 69]. Immune-histochemical staining were performed according to previously reports [70]. In short, tissues were treated with 0.3% H_2_O_2_ and block with normal serum, and then incubated with primary antibodies against CD163 (GB11340-1, Srvicebio) and Mrc1 (ab64693, Abcam), or non-immune serum for negative control overnight at 4 ℃. After washing and incubated with a biotinylated secondary antibody, the slides were applied to react with the avidin–biotin complex reagent conjugated with horseradish peroxidase, and then counterstained with hematoxylin and mounted for examination under a microscope.

### Statistical analysis

Statistical analysis were presented as mean ± SEM. To determine the statistical significance, *P* values were generated using one-way ANOVA with the Bonferroni correction or unpaired t-test with *P <* 0.05 representing a statistically significant difference. The significance is shown compared with the control group.

## Supplementary Information

**Additional file 1:** **Figure S1.** t-SNE map of cell populations of the rat kidney.

**Additional file 2:** **Figure S2.** Expression features of differential expressed genes in the bulk kidney of calauli rats.

**Additional file 3:** **Figure S3. **Expression features of differential expressed genes in the bulk kidney of calauli rats.

**Additional file 4: Figure S4. **Distinct injured-proximal tubule clusters in rats with calculi.

**Additional file 5: Figure S5. **Expression features and functional enrichments of proximal tubular subpopulations.

**Additional file 6:** **Figure S6. **Expression features of distinct markers in BI subclusters.

**Additional file 7:** **Figure S7. **KEEG enrichments of DEGs in the BI clusters in kidneys of rats with calculi.

**Additional file 8** Supplementary Methods.

## Data Availability

The raw sequence data reported in this paper have been deposited in the Genome Sequence Archive [71] in National Genomics Data Center [72], China National Center for Bioinformation/Beijing Institute of Genomics, Chinese Academy of Sciences (GSA: CRA010121) that are publicly accessible at https://ngdc.cncb.ac.cn/gsa.

## Abbreviations

AKI: Acute kidney injury
CaOx: Calcium oxalate
COM: Calcium oxalate monohydrate
PT: Proximal tubule
PCT: Proximal convoluted tubule
PST: Proximal straight tubule segments
snRNA-seq: Single nucleus RNA sequencing
ST: Spatial transcriptomics
t-SNE: t-distributed stochastic neighbor embedding
UMAP: Uniform manifold approximation and projection
BI: Blood and immune
NK: Natural killer
DEG: Differentially expressed genes
KEGG: Kyoto Encyclopedia of Genes and Genomes
GSEA: Gene set enrichment analysis
TRP: Transient receptor potential
GEM: Gel beads-in-emulsion
PCR: Polymerase chain reaction
HE: Hematoxylin-eosin
GO: Gene ontology
MF: Molecular function
CC: Cellular component
BP: Biological process
ESRD: End-stage renal failure.
