# Supplementary figures and images for: Integrated single-nucleus sequencing and spatial architecture analysis identified distinct injured-proximal tubular types in calculi rats
Source: Cell Biosci. 2023 May 19;13:92. doi: 10.1186/s13578-023-01041-3 (PMC10197242; doi:10.1186/s13578-023-01041-3)

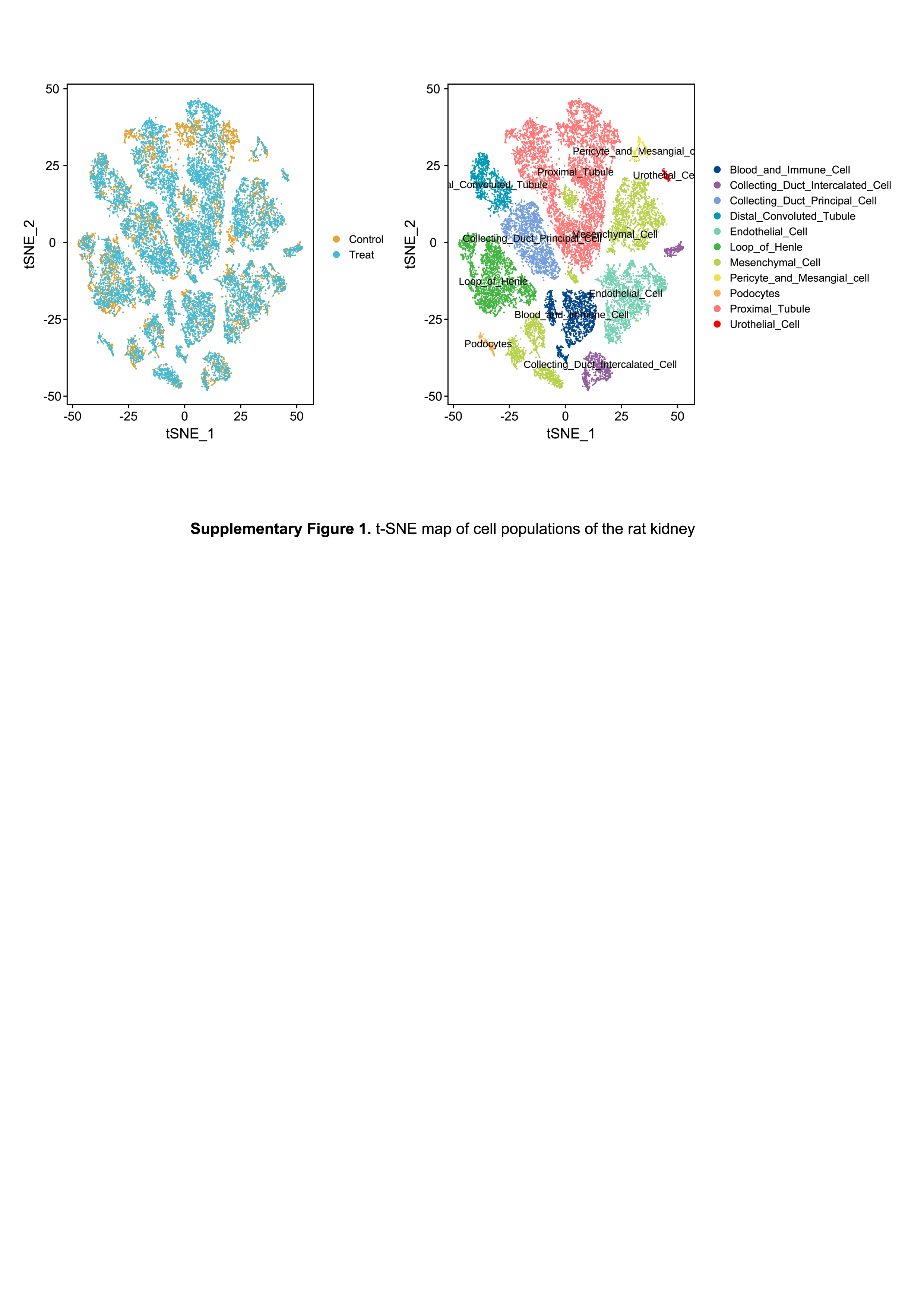

Supplement: Supplementary file 1 — Additional file 1: Figure S1. t-SNE map of cell populations of the rat kidney. [file 13578_2023_1041_MOESM1_ESM.tif]

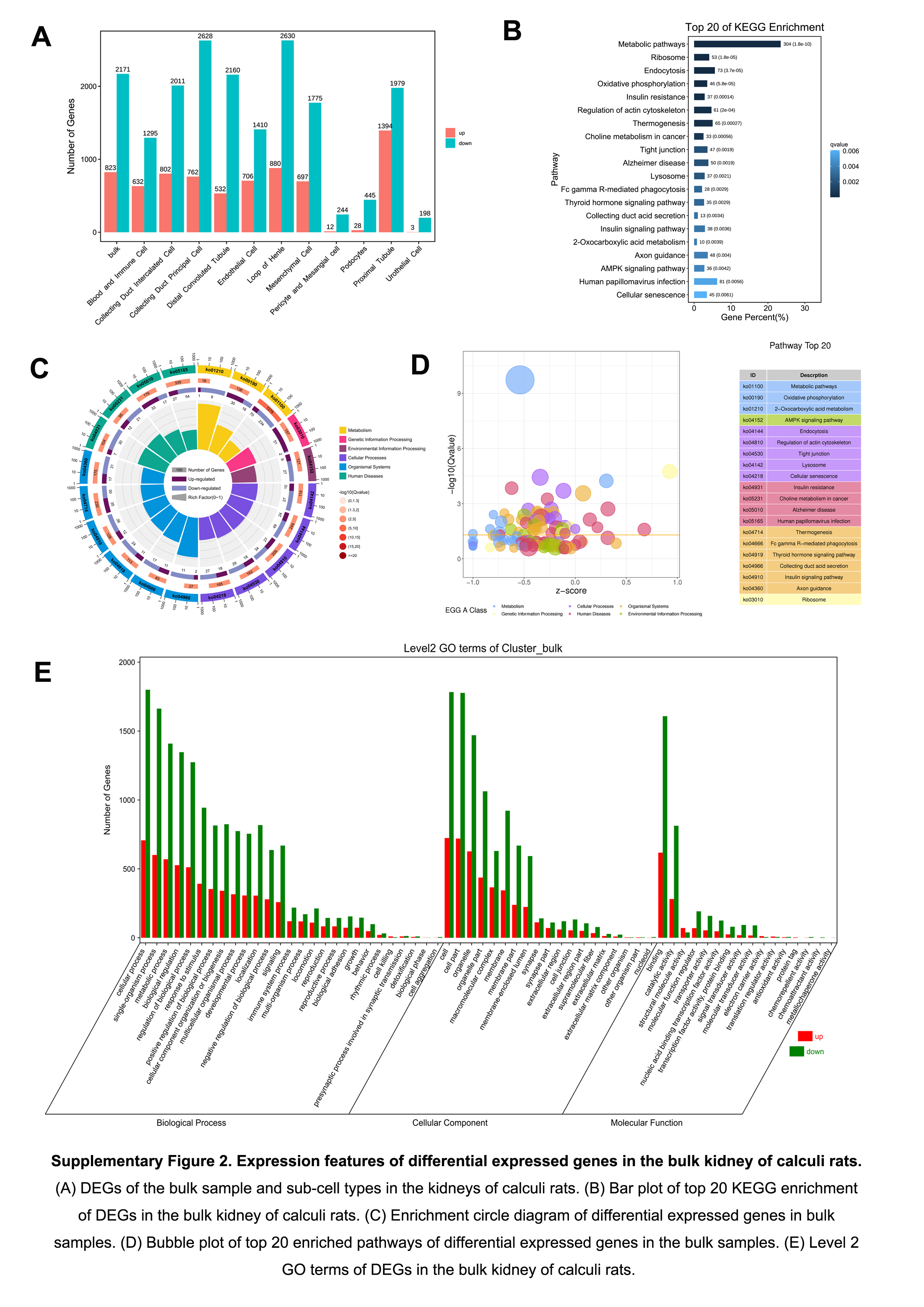

Supplement: Supplementary file 2 — Additional file 2: Figure S2. Expression features of differential expressed genes in the bulk kidney of calauli rats. [file 13578_2023_1041_MOESM2_ESM.tif]

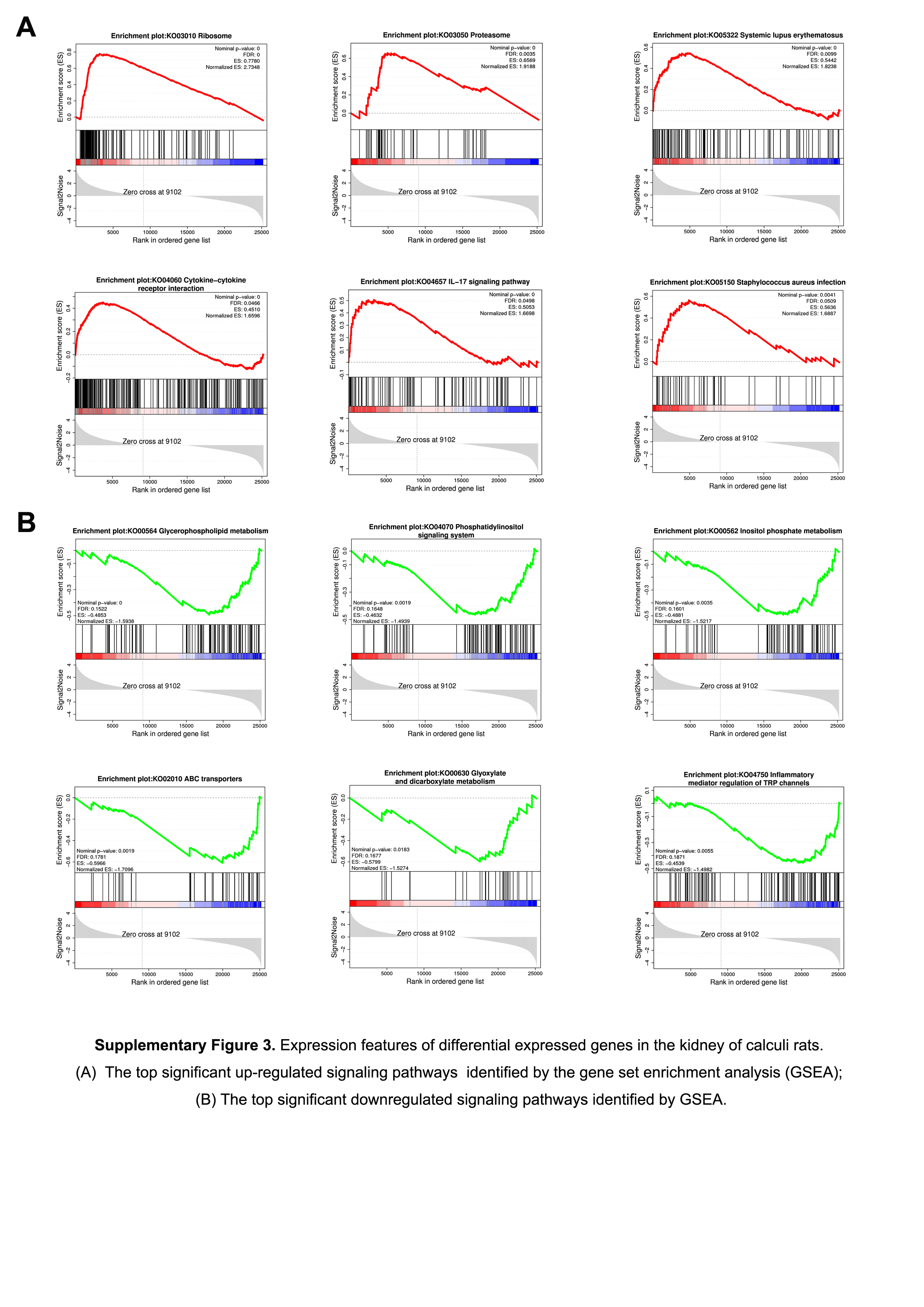

Supplement: Supplementary file 3 — Additional file 3: Figure S3. Expression features of differential expressed genes in the bulk kidney of calauli rats. [file 13578_2023_1041_MOESM3_ESM.tif]

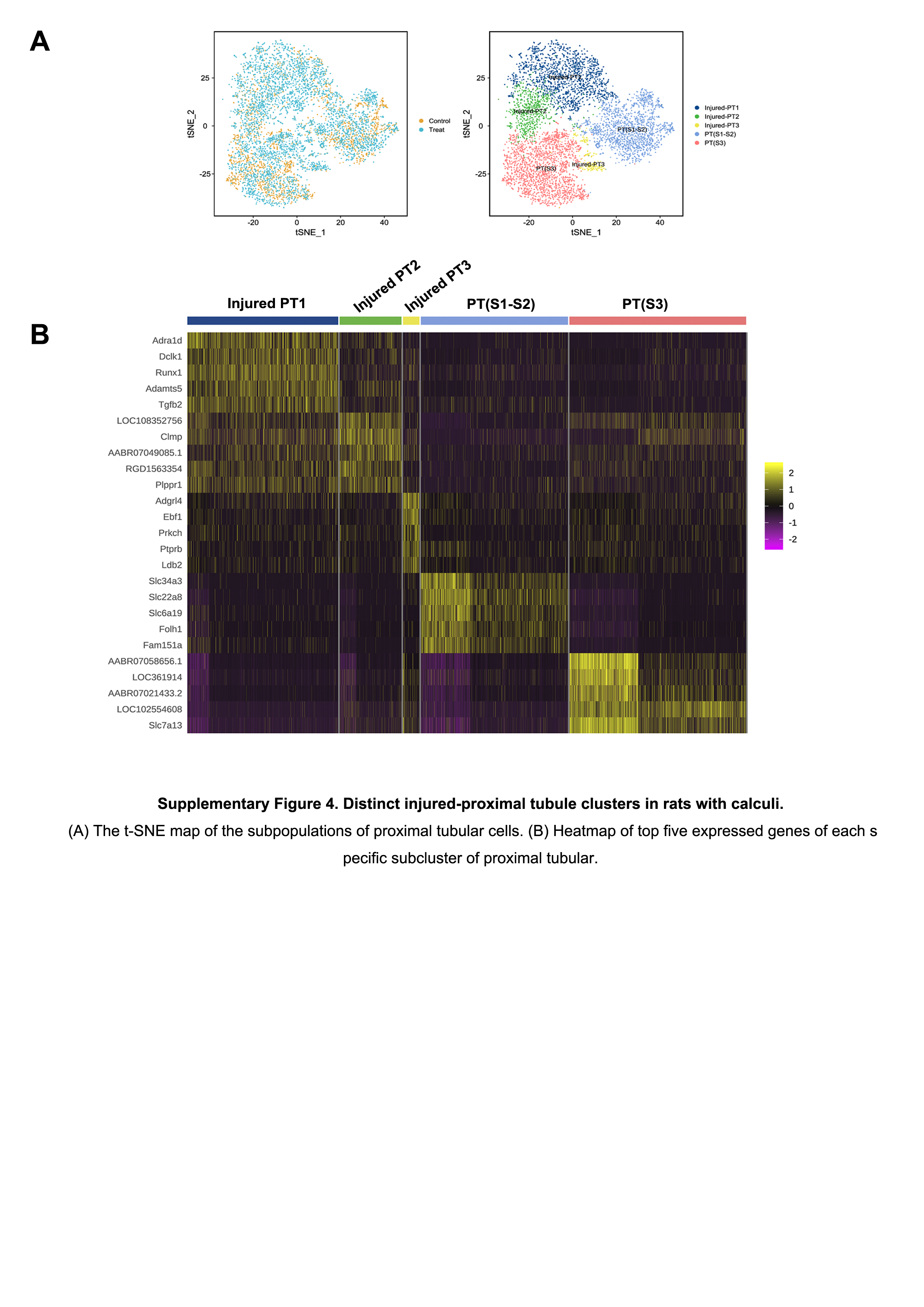

Supplement: Supplementary file 4 — Additional file 4: Figure S4. Distinct injured-proximal tubule clusters in rats with calculi. [file 13578_2023_1041_MOESM4_ESM.tif]

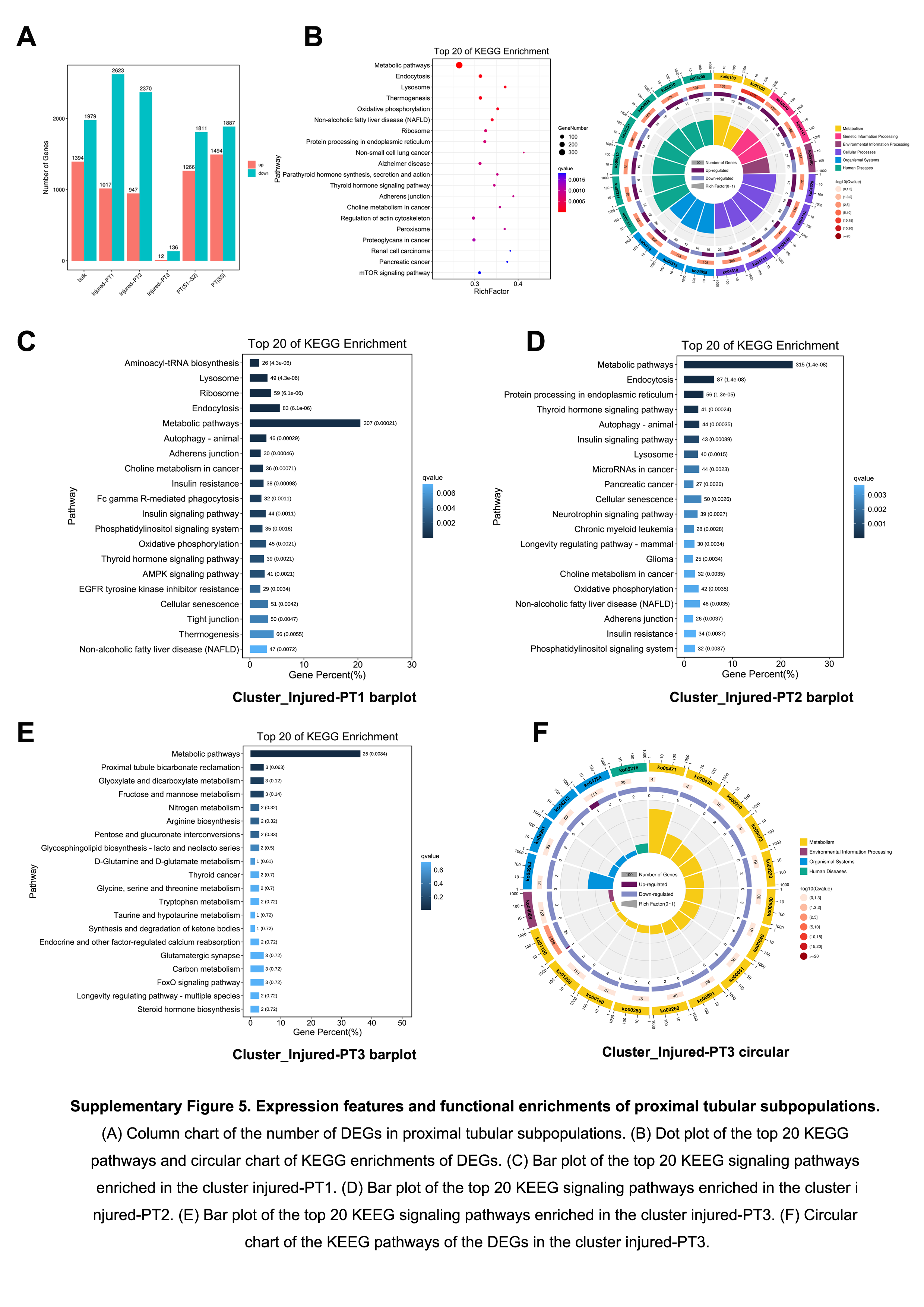

Supplement: Supplementary file 5 — Additional file 5: Figure S5. Expression features and functional enrichments of proximal tubular subpopulations. [file 13578_2023_1041_MOESM5_ESM.tif]

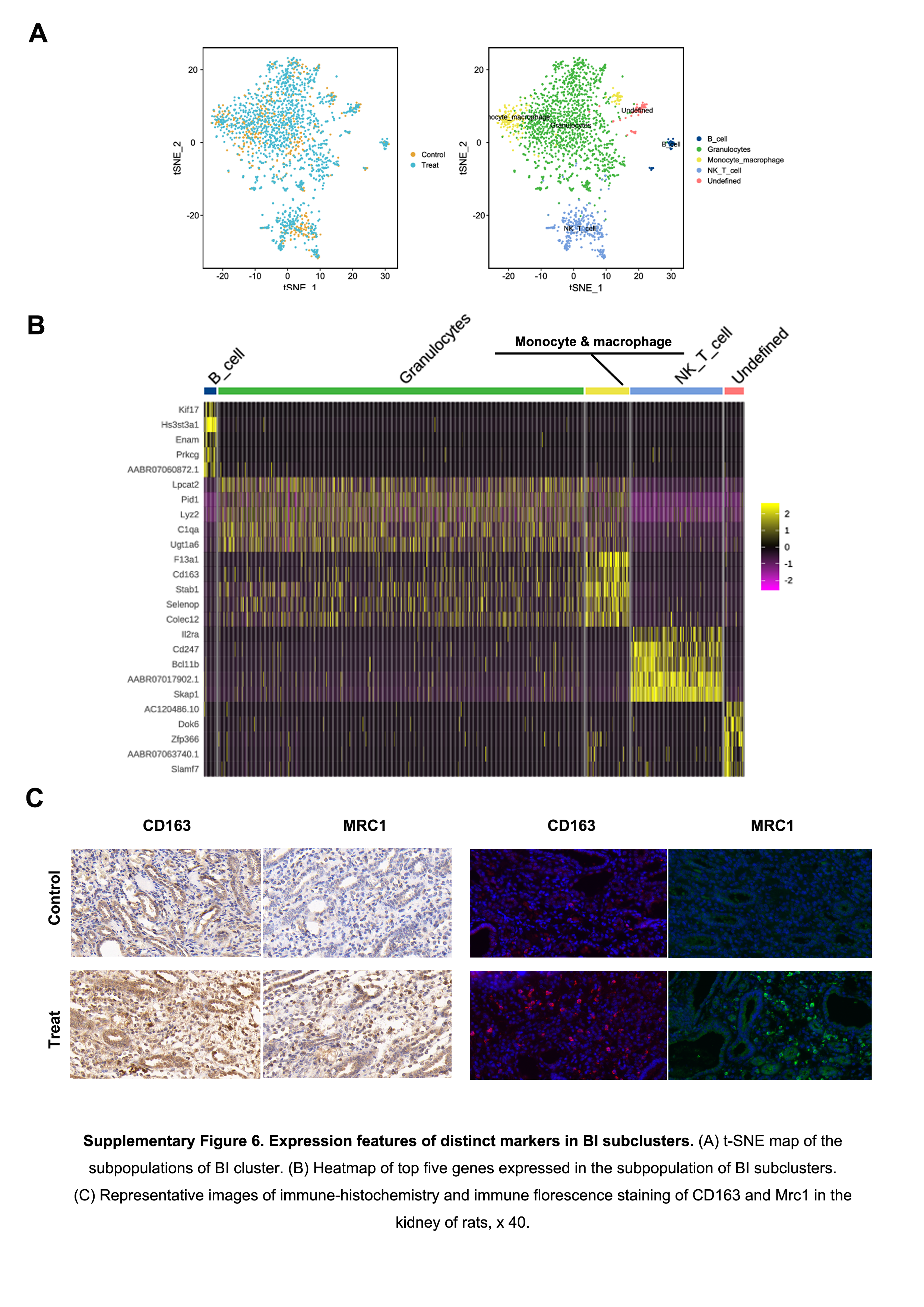

Supplement: Supplementary file 6 — Additional file 6: Figure S6. Expression features of distinct markers in BI subclusters. [file 13578_2023_1041_MOESM6_ESM.tif]

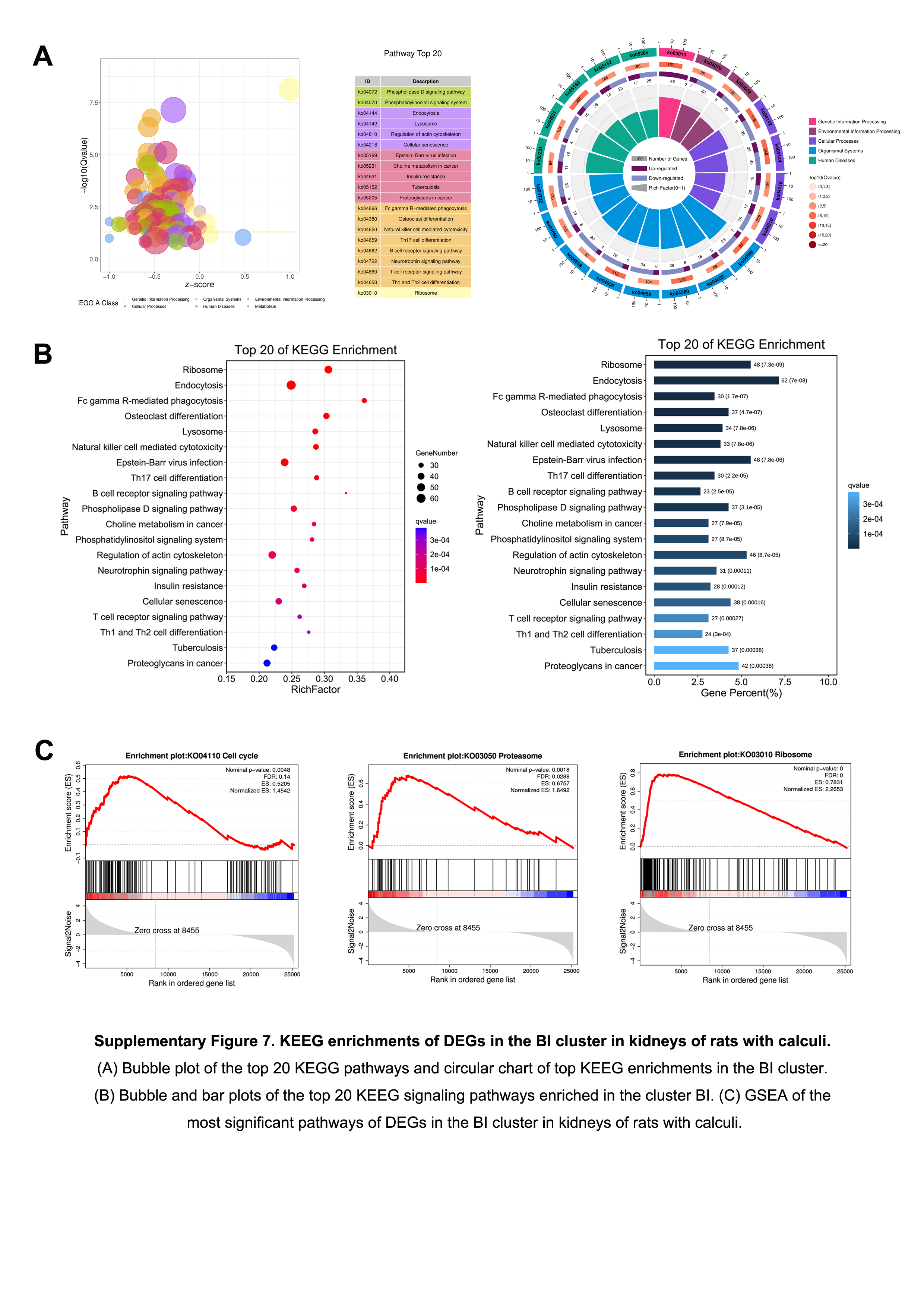

Supplement: Supplementary file 7 — Additional file 7: Figure S7. KEEG enrichments of DEGs in the BI clusters in kidneys of rats with calculi. [file 13578_2023_1041_MOESM7_ESM.tif]
