# Supplementary material for: Integrated single-nucleus sequencing and spatial architecture analysis identified distinct injured-proximal tubular types in calculi rats
Source: Cell Biosci. 2023 May 19;13:92. doi: 10.1186/s13578-023-01041-3 (PMC10197242; doi:10.1186/s13578-023-01041-3)
Supplement: Supplementary file 8 — Additional file 8 Supplementary Methods. [file 13578_2023_1041_MOESM8_ESM.docx]

**Integrated single-nucleus sequencing and spatial architecture analysis identified distinct injured-proximal tubular types in calculi rats**

**Supplementary methods**

**1. snRNA sequencing data processing and analysis**

**1.1 Single Nuclei Isolation**

Nuclei were isolated using Iodoxanol density gradient centrifugation. Samples cut into pieces were homogenized using a Dounce homogenizer in 2 ml of ice-cold Nuclei Lysis Buffer (0.25 M sucrose, 5 mM CaCl_2_, 3mM MgAc_2_, 10 mM Tris-HCl pH 8.0, 1mM DTT, 0.1 mM EDTA, Protease Inhibitor, 1U/ul RiboLock RNase Inhibitor). The homogenate was filtered through a 70 μm cell strainer, and then equal volume of 50% Iodoxanol (0.16 M sucrose, 10 mM NaCl, 3 mM MgCl_2_, 10 mM Tris-HCl pH 7.4, 1U/μl RiboLock RNase Inhibitor, 1 mM DTT, 0.1 mM PMSF Protease Inhibitor, suitable volume of Iodoxanol) was added to the filtrate to make the final concentration of Iodoxanol at 25%. 33% and 30% Iodoxanol were added in to 15 ml tube slowly in sequence for the preparation of a gradient solution. After the addition of the 25% Iodoxanol with nuclei, the solution was centrifuge at 3234 g for 20 min at 4℃. Solutions under the 30% Iodoxanol liquid level were transfer to a new tube, and washed with Nuclei Wash Buffer (PBS with 0.04% BSA, 0.2U/ul RiboLock RNase Inhibitor, 500 mM mannitol, 0.1mM PMSF Protease Inhibitor). The pellet was resuspended and washed twice with the Nuclei wash buffer. After another centrifugation, the pellet was resuspended in Nuclei Wash Buffer, filtered through a 40 μm cell strainer, and counted.

**1.2 GEM generation & Barcoding**

Briefly, Master mix was prepared on ice. Appropriate volume of nuclease free water and corresponding volume of single cell suspension were added to Master Mix for a total of 75 µl

in each tube, and 70 µl was loaded in Chip wells (Row 1). 50 µl Gel Beads was aspirated slowly into the wells (Row 2). 45 µl Partitioning Oil was dispensed into the wells in row labeled 3. Attach 10X gasket, and run the Chromium Controller. Slowly aspirate 100 µl Gel Beads-In-Emulsions (GEMs) from the lowest points of the recovery wells in the top row labeled 3, dispense GEMs into the tube strip on ice. Upon dissolution of the Single Cell 3’ Gel Bead in a GEM, primers containing (i) an Illumina® R1 sequence (read 1 sequencing primer), (ii) a 16 nt 10x Barcode, (iii) a 10 nt Unique Molecular Identifier (UMI), and (iv) a poly-dT primer sequence are released and mixed with cell lysate and Master Mix. GEM-RT incubation was performed in a thermal cycler with the following steps: 53℃, 45 min; 85℃, 5 min; 4℃, hold. Incubation of the GEMs then produces barcoded, full-length cDNA from poly-adenylated mRNA. After incubation, the GEMs are broken and the pooled fractions are recovered.

**1.3 GEM-RT cleanup & cDNA amplification**

Silane magnetic beads (10 X Genomics, PN-2000048) are used to remove leftover biochemical reagents and primers from the post GEM reaction mixture. Full-length, barcoded cDNA is then amplified by PCR to generate sufficient mass for library construction. cDNA is clean up using SPRIselect Reagent Kit (Beckman Coulter B23318). cDNA quality control and quantification were determined on Agilent Bioanalyzer High Sensitivity chip.

**1.4 Library construction**

R1 (read 1 primer sequence) are added to the molecules during GEM incubation. P5, P7, a sample index, and R2 (read 2 primer sequence) are added during library construction via End Repair, A-tailing, Adaptor Ligation, and PCR. The final libraries contain the P5 and P7 primers used in Illumina bridge amplification. Library construction quality control was run on an Agilent Bioanalyzer High Sensitivity chip.

**1.5 Sequencing**

ABI StepOnePlus Real-Time PCR System (Life Technologies) was applied for quantitative analysis and pooling, sequencing was performed according to the PE150 mode of Novaseq 6000. The Single Cell 3’ Protocol produces Illumina-ready sequencing libraries. A Single Cell 3’ Library comprises standard Illumina paired-end constructs which begin and end with P5 and P7. 16 bp 10X Barcode and 10 bp UMI are encoded in Read 1, while Read 2 is used to sequence the cDNA fragment. Sample index sequences are incorporated as the i7 index read. Read 1 and Read 2 are standard Illumina® sequencing primer sites used in paired-end sequencing.

**1.6 Data processing**

Raw sequencing data was processed by Cell Ranger (10× Genomics, version 3.0.2) pipeline and aligned to the rat reference genome (Ensembl_release100.Rnor_6.0). After the UMI correction and count, we obtained the unfiltered feature barcode matrix. Based on the unfiltered feature barcode matrix, the data from cells or non-cell was identified and distinguished by Cell Ranger, a rank plot was applied to visually reflect the results of the identified effective cells.

Briefly, reads with low-quality barcodes and UMIs were filtered out and then mapped to the reference genome. Reads uniquely mapped to the transcriptome and intersecting an exon at least 50% were considered for UMI counting. Before quantification, the UMI sequences would be corrected for sequencing errors, and valid barcodes were identified based on the EmptyDrops method [1]. The cell by gene matrices were produced via UMI counting and cell barcodes calling. The cell by gene matrices for each sample were individually imported to Seurat [2] version 3.1.1 for downstream analysis. Details regarding the Seurat analyses performed in this study can be also found in the website tutorial (https://satijalab.org/seurat/v3.0/pbmc3k_tutorial. html).

**1.6.1 Identification of cell types and marker genes**

Cells with unusually high number of UMIs (≥8000) or mitochondrial gene percent (≥10%) were filtered out. We also excluded cells with less than 500 or more than 4000 genes detected. After removal of low-quality cells, we used harmony [3] for data consolidation and batch effect correction. Clusters were visualized using the Uniform Manifold Approximation and Projection (UMAP). Cell type identities were characterized based on the expression of known markers in the Cell Marker database and reported studies. Based on the classification results of cell subpopulations, the classification results of single cell subpopulations are visualized by using tSNE (t-distributed stochastic neighbor embedding) nonlinear clustering method [4].

**1.6.2 Differentially expressed genes analysis**

Expression value of each gene in given cluster were compared against the rest of cells using Wilcoxon rank sum test [8]. Significant upregulated genes were identified using a number of criteria. First, genes had to be at least 1.28-fold overexpressed in the target cluster. Second, genes had to be expressed in more than 25% of the cells belonging to the target cluster. Third, p value is less than 0.05.

GO enrichment analysis provides all GO terms that significantly enriched in differentially expressed genes comparing to the genome background and filter the differentially expressed genes that correspond to biological functions. Firstly, all peak related genes were mapped to GO terms in the Gene Ontology database (http://www.geneontology.org/), gene numbers were calculated for every term, significantly enriched GO terms in differentially expressed genes comparing to the genome background were defined by hypergeometric test. KEGG Pathway enrichment analysis identified significantly enriched metabolic pathways or signal transduction pathways in differentially expressed genes comparing with the whole genome background. The calculated p-value were gone through FDR Correction, taking FDR ≤ 0.05 as a threshold. Enrichment analysis was performed on omicshare. KEGG: https://www.omicshare.com/tools/Home/Soft/pathwaygseasenior

**1.6.3 Gene set enrichment analysis (GSEA)**

Gene set enrichment analysis was performed with the R package fgsea with default parameters. Genes were ranked within clusters by multiplying avg_logFC by –log_10_(p_val) obtained from comparing stone and control samples with the FindMarkers function in Seurat.

**1.6.4 Ligand-receptor interaction analysis**

To study ligand-receptor interactions, we used a draft network published by Ramilowski et al [5]. We examined proximal tubular or blood and immune cell types and required that 1) the ligand, receptor, or both were differentially expressed and 2) its cognate pair was expressed in the partner cell type.

**2. Sample preparation and spatial transcriptomics**

**2.1 Sample preparing**

The embedded tissue blocks are cryosectioned in a cryostat to generate 10 μm sections for Visium Spatial slides while keeping the samples frozen. Sections are placed respectively on Visium Spatial Tissue Optimization Slide and Visium Spatial Slide within the capture area. HE staining and microscope bright field imaging for sections are then processed.

**2.2 Tissue Optimization**

The Visium Spatial Tissue Optimization Slide kit (10X Genomics, PN-1000191) is used to fit the time for permeabilization by generating fluorescently labeled cDNA tissue prints. A timer gradient (3 min, 6 min, 12 min, 18 min,24 min, and 30 min) is set for each capture area. Fluorescent cDNA synthesis is performed and fluorescent print of spatial positions where the cDNA reaction took place. The fluorescent print is imaged using fluorescence microscope with tissue removed. The section with 18 min of permeabilization has the strongest fluorescence signal, minimum diffusion and longest time for permeabilization, and 18 min is chosen as the most suitable time for permeabilization.

**2.3 Permeabilization and cDNA amplification**

Permeabilization processes for the time determined by tissue optimization. The first strand of cDNA is synthesized via reverse transcription and the second strand of cDNA is synthesized via PCR. Then the cDNA is denaturation, making the second strand of cDNA dissociated from slide. The spatially barcoded, full-length cDNA is amplified via PCR to generate sufficient mass for library construction.

**2.4 Library construction**

Enzymatic fragmentation and size selection are used to optimize the cDNA amplicon size. P5, P7, i7 and i5 sample indexes, and TruSeq Read 2 (read 2 primer sequence) are added via End Repair, A-tailing, Adaptor Ligation, and PCR. The final libraries contain the P5 and P7 primers used in Illumina amplification.

**2.5 Sequencing**

ABI StepOnePlus Real-Time PCR System (Life Technologies) was applied for quantitative analysis and pooling, sequencing was performed according to the PE150 mode of Novaseq 6000: Read 1, 28 cycles; Read 2, 120 cycles; i5 index, 10 cycles; i7 index, 10 cycles.

The Visium Spatial protocol produces Illumina-ready sequencing libraries. A Visium Spatial library comprises standard Illumina paired-end constructs which begin and end with P5 and P7. The Visium Spatial 16 bp spatial barcode and 10 bp UMI are encoded in Read 1, while Read 2 is used to sequence the cDNA fragment. Sample index sequences are incorporated as the i7 index read. Read 1 and Read 2 are standard Illumina® sequencing primer sites used in paired-end sequencing.

**2.6 Data processing**

Raw sequencing data was processed by Space Ranger (10× Genomics) and aligned to the rat reference genome (Ensembl_release100.Rnor_6.0). Raw data, tissue staining, as well as Chip serial numbers were input into space ranger for data quality control and sequence alignment to obtain high-quality sequencing data, the spatial distribution of spots in the tissue and the expression of genes in each spot.

Data was performed log homogenization, and PCA analysis was employed to reduce the variables, spots were clustered and classified by the graph-based clustering algorithm. Spots were visualized using tSNE, and mapped in the tissue according to the staining result to view the distribution of each subgroup.

**References**

1. Lun ATL, Riesenfeld S, Andrews T, Dao TP, Gomes T, participants in the 1st Human Cell Atlas J, Marioni JC: **EmptyDrops: distinguishing cells from empty droplets in droplet-based single-cell RNA sequencing data**. *Genome Biol* 2019, **20**(1):63.

2. Butler A, Hoffman P, Smibert P, Papalexi E, Satija R: **Integrating single-cell transcriptomic data across different conditions, technologies, and species**. *Nat Biotechnol* 2018, **36**(5):411-420.

3. Korsunsky I, Millard N, Fan J, Slowikowski K, Zhang F, Wei K, Baglaenko Y, Brenner M, Loh PR, Raychaudhuri S: **Fast, sensitive and accurate integration of single-cell data with Harmony**. *Nat Methods* 2019, **16**(12):1289-1296.

4. Kobak D, Berens P: **The art of using t-SNE for single-cell transcriptomics**. *Nat Commun* 2019, **10**(1):5416.

5. Ramilowski JA, Goldberg T, Harshbarger J, Kloppmann E, Lizio M, Satagopam VP, Itoh M, Kawaji H, Carninci P, Rost B *et al*: **A draft network of ligand-receptor-mediated multicellular signalling in human**. *Nat Commun* 2015, **6**:7866.
